# Diagnosis of Small Intestinal Diaphragms and Strictures Induced by Non-steroidal Anti-inflammatory Drugs Through Intraoperative Enteroscopy: A Case Study From Saudi Arabia

**DOI:** 10.7759/cureus.59752

**Published:** 2024-05-06

**Authors:** Rafaat M Chakik, Nasser I Alqahtani, Yahia Al-Hagawi, Saeed Nasser Alsharif, Abdullah S Alqahtani, Dawlah Hadi Asiri, Salihah Y Al-Mani

**Affiliations:** 1 Gastroenterology, Armed Forces Hospital Southern Region (AFHSR), Khamis Mushait, SAU; 2 Colorectal Surgery, Armed Forces Hospital Southern Region (AFHSR), Khamis Mushait, SAU; 3 Internal Medicine, Armed Forces Hospital Southern Region (AFHSR), Khamis Mushait, SAU

**Keywords:** post operative histopathology, nsaid abuse, colonoscopy, esophagogastroduodenoscopy (egd), small intestinal diaphragms

## Abstract

The small intestinal diaphragms are a rare condition characterized by focal or diffuse luminal narrowing in the small intestine. Nonsteroidal anti-inflammatory drugs (NSAIDs) have been associated with the development of small intestinal diaphragms, particularly in long-term and high-dose users. In the current report, a Saudi adult female complained of abnormal bowel motions, which caused severe abdominal pain. She had long-term treatment with NSAID. Systemic and physical examination was unremarkable, apart from poorly localized, nonspecific tenderness to abdominal palpation. Two endoscopic investigations (upper and lower endoscopy) were performed to identify any abnormalities in the digestive system; also, a biopsy was taken for histopathological analysis. In addition, another capsule endoscopy was done to investigate any abnormal bowel motion. The findings of two endoscopic investigations and histopathological analysis of duodenum biopsies revealed different features of small intestinal diaphragms and stricture. The biopsies showed mild chronic inflammation. The esophagogastroduodenoscopy (EGD) and colonoscopy showed multiple strictures and ulcerations in the small bowel. Also, a diffused mucosal erythema of the stomach and a remarkable scar on the third part of the duodenum were detected. That might be due to the excessive use of NSAIDs. The investigations revealed multiple small bowel diaphragmatic stenosis and strictures in the proximal and distal small bowel. These are distinct signs of NSAID-induced small bowel diaphragms and strictures.

## Introduction

Small bowel diaphragm usually refers to a rare disorder called diaphragm disease or intestinal diaphragm disease. This disorder is characterized by thin membrane strictures, or diaphragms, in the small intestine [1]. These diaphragms can cause partial or complete bowel blockage, resulting in symptoms such as abdominal discomfort, bloating, nausea, vomiting, and changes in bowel habits [2]. The specific etiology of small bowel diaphragm disease is unknown; however, it is thought to be linked to persistent intestinal inflammation similar to that observed in Crohn's disease. Other possible reasons are ischemia (limited blood supply to the colon) and certain medicines, notably nonsteroidal anti-inflammatory drugs (NSAIDs) [2]. Small bowel diaphragm disease is often diagnosed via imaging techniques such as CT scans, MR enterorrhaphy, or small bowel follow-through examinations. One of the treatment options for the small bowel is the use of double-balloon enteroscopy (DBE), which facilitates the assessment and management of small bowel strictures caused by intrinsic or extrinsic factors; it also addresses bleeding and polyps [3]. Corticosteroids and immunosuppressants may be used to treat inflammation and symptoms [4]. In some circumstances, surgical intervention may be required to remove the strictures and relieve the blockage [5].

The underlying cause of small bowel disease induced by NSAIDs implies that heightened intestinal permeability serves as the pivotal mechanism for converting biochemical harm into tissue damage, which has been reported in almost 70% of patients with different degrees [6,7]. Previous studies revealed that NSAIDs downregulate the expression levels of cyclooxygenase-1, which might cause a delay in microcirculation, ulcers, ischemia, strictures, and mucosal diaphragms [6-8]. Furthermore, NSAIDs can impede mucosal repair, worsen existing injuries, and encourage the creation of strictures or diaphragms in the small intestine. These diaphragms can cause partial or complete bowel blockage, leading to symptoms such as abdominal discomfort, bloating, and changes in bowel habits [9]. Overall, NSAIDs contribute to the development of small bowel diaphragm disease by causing inflammation, increasing intestinal permeability, and disrupting mucosal repair mechanisms.

In the current research, we reported a Saudi patient diagnosed with a typical small bowel diaphragm, which is thought to be associated with long-term exposure to NSAIDs.

## Case presentation

A 25-year-old Saudi female presented to the emergency department of the Armed Forces Hospital, Southern Region, Khamis Mushait City, Saudi Arabia, in June 2022 with a complaint of continuous abdominal pain for three days. The patient was in her usual condition until three days before she presented to the emergency room with a history of diffuse abdominal pain (gradual onset, progressive in course, not radiated to any site, and without any reliving or aggravating factors), with two to three bowel motions per day, and no association with mucus or blood. She had neither dysphagia, odynophagia, vomiting, jaundice, hematemesis, melena, tenesmus, nor fever, with slight weight loss. She has been using NSAIDs, including diclofenac, three times per day every two days for two months as a secondary treatment for her toothache in the last two years. There was no history of uveitis, scleritis, episcleritis, oral or genital ulcers, joint pain, skin rash, or weight loss. The patient denied any previous history of respiratory, cardiac, or neurologic symptoms. Also, no past surgical or medical history with the same presentation was reported before. There were no reports of any past medical history for the same presentation in the family or mention of any recent travel, smoking, alcoholic consumption, illegal drugs, or eating out. She was living in Khamis Mushait with her family.

The primary physical examination upon admission demonstrated a normal blood pressure of 122/63 mmHg (mean arterial pressure of 68), a heart pulse rate of 89 per minute, oxygen saturation of 96% in room air, a respiratory rate of 19 breaths per minute, and a body temperature of 36.4° C. Her face was not pale or jaundiced, without any conjunctivitis, rashes, or oral ulcers, and not dehydrated. Besides, no signs of hypopigmentation or hyperpigmentation were noticed.

The neck showed no signs of palpable lymph nodes or dilated veins. No signs of clubbing, cyanosis, or palpable lymph nodes in the neck, axilla, or inguinal were seen as well. There were no skin lesions throughout the body. The abdominal examination was unremarkable, apart from diffuse mild abdominal pain. The auscultation didn't reveal any renal bruit or abnormal bowel sounds. The rectal examination was unremarkable regarding the melena, perianal fissures, fistulas, or abscess symptoms; however, they were reported in the history.

Table 1 demonstrates laboratory tests done to investigate her condition. As seen, most of the tests had normal levels except for platelet (Plt) count and erythrocyte sedimentation rate (ESR), prothrombin time (Pt), and international normalized ratio (INR), which were higher than normal levels. Also, the results showed slightly lower levels of hemoglobin (Hb), mean corpuscular volume (MCV), ferritin, serum creatinine (sCr), and phosphorus (Ph), and a borderline decrease in the levels of gamma-glutamyl transpeptidase (GGT).

**Table 1:** The first blood analysis of the studied case

Variables	Results	Normal values
Hematology		
White blood cells (WBC), ×10^9^/L	9.58	4-11
Hemoglobin (Hb), g/dL	11.9 ↓	12-15.1
Hematocrit (HCT), %	41	38-52
Platelets count (Plt), ×10^9^/L	528 ↑	150-400
Mean corpuscular volume (MCV), fl	64 ↓	80-100
Neutrophils absolute count, ×10^9^/L	4.5	2-7.5
Lymphocytes absolute count, ×10^9^/L	3.8	1.5-4
Eosinophiles absolute count, ×10^9^/L	0.2	0.1-0.5
Monocytes absolute count, ×10^9^/L	0.7	0.2-1
Erythrocyte sedimentation rate (ESR), mm/hr	36 ↑	<15.5
Liver function and comprehensive metabolic panel (CMP)		
C-reactive protein (CRP), mg/L	7.5	<10
Ferritin, ng/mL	10 ↓	12-150
Magnesium (Mg), mmol/L	0.83	0.65-1.05
Lipase, U/L	55	0-160
Total bilirubin (Tb), µmol/L	6	5.1-20.5
Direct bilirubin (Db), mg/dL	3.1	1.7-8.6
Alanine transaminase (ALT), U/L	14	7-55
Aspartate transaminase (AST), U/L	30	8-48
Alkaline phosphatase (ALP), U/L	66	32-91
Gamma-glutamyl transpeptidase (GGT), IU/L	11	11-55
Prothrombin time (Pt), seconds	15.8 ↑	11-13
Activated partial thromboplastin time (aPTT), seconds	30.8	21-35
International normalized ratio (INR)	1.2 ↑	<1.1
Kidney functions and basic metabolic panel		
Sodium (Na), mmol/L	138	136-144
Potassium (K), mmol/L	3.9	3.6-5.1
Calcium (Ca), mmol/L	2.37	2.2-2.7
Chloride (Cl), mmol/L	105	96-106
Serum creatinine (sCr), µmol/L	40 ↓	53-97.2
Blood urea nitrogen (BUN), mmol/L	4.1	2.1-8.5
Phosphorus (Ph), mg/dL	1.13 ↓	2.5-4.5

**Figure 1:**
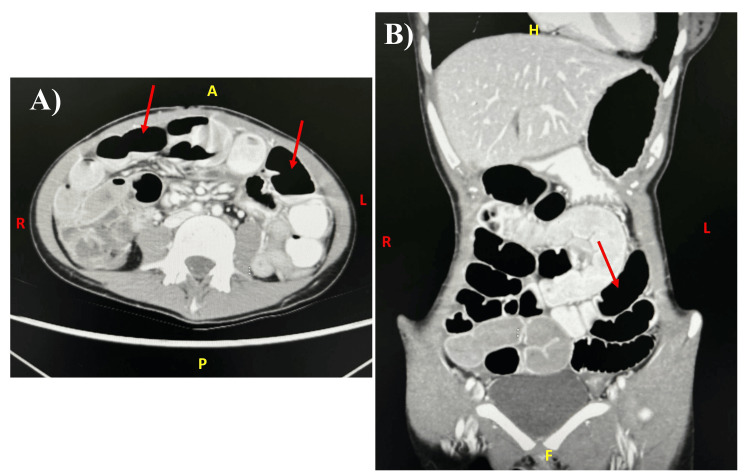
The abdominal computed tomography (CT) of the studied case CT showed a diffuse dilatation of the small bowel loops, which affected most of the jejunum and ileum (red arrows). A: axial view; B: coronal view

Before admission, the patient was assessed by the general surgery team, who requested a computed tomography (CT) scan of the abdomen with contrast. The CT showed a diffuse dilatation of the small bowel loops; the maximum dimension was 5 cm, which affected most of the jejunum and ileum; however, the terminal ileum was not dilated. Also, there were associated air-fluid levels and minimal free fluid between the dilated pelvic lobes. The small bowel loops were presenting prominent diffuse wall enhancement and variable relative diffuse thickening. The bowel wall thickening and enhancement were also seen to affect the colon. All of these features were suggestive of inflammatory bowel disease (IBD) with non-mechanical obstruction (ileus). Furthermore, there were significantly enlarged mesenteric lymph nodes, with the largest measuring about 2.7×1.1 cm (Figure 1).

The patient was admitted to the gastroenterology team as a case of IBD. Further laboratory examinations were requested. The results showed negative hepatic serology (hepatitis B surface antigen, HBsAg, and hepatitis C virus, HCV), anti-tissue transglutaminase IgG, and anti-tissue transglutaminase IgA. Furthermore, the stool culture and Clostridium difficile test (for ova and parasites) were all negative. The thyroid function and antinuclear antibody (ANA) tests were normal. The results showed a remarkable deficiency in the levels of vitamin B-12 (101 pg/mL).

**Figure 2:**
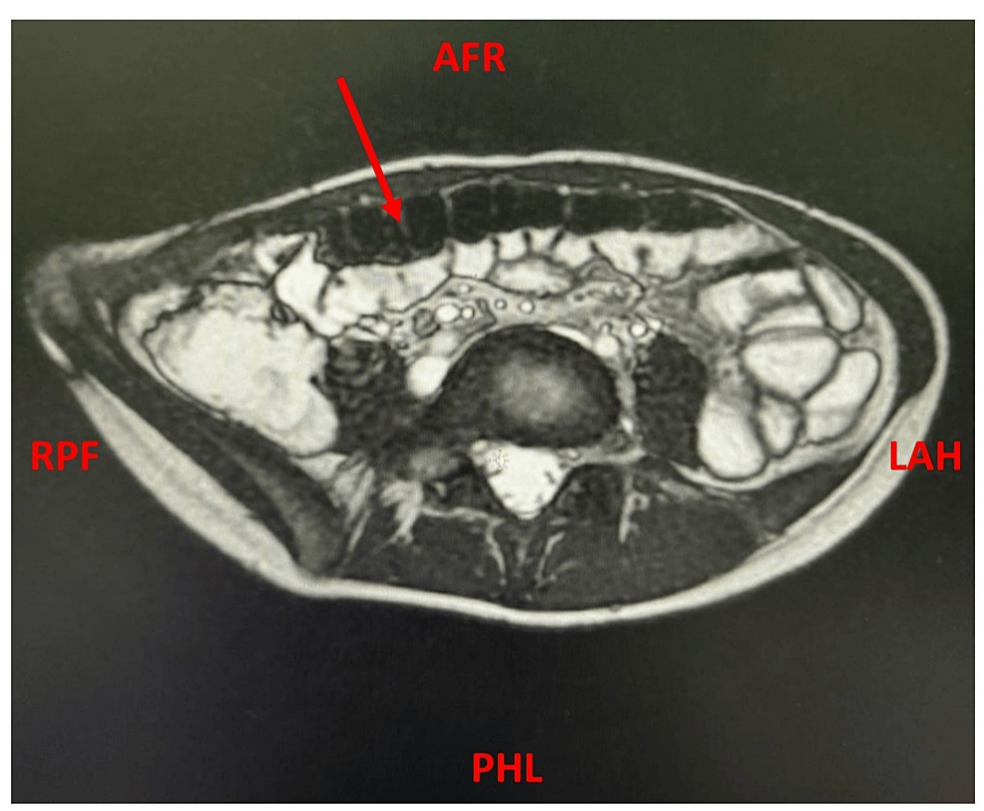
The magnetic resonance imaging (MRI) enterography of the studied patient The red arrow indicates abnormal bowel motion. AFR - anterior-foot-right; PHL - posterior-head-left; RPF - right-posterior-foot; LAH - left-anterior-head

Furthermore, a magnetic resonance imaging (MRI) enterography was performed, with oral and intravenous contrast. MRI enterography revealed an extensive bowel motion of 5 cm and artifacts with normal fold distribution of the jejunum and ileum. No suspicious, clear focal lesions or obstructing lesions were detected (Figure 2).

**Figure 3:**
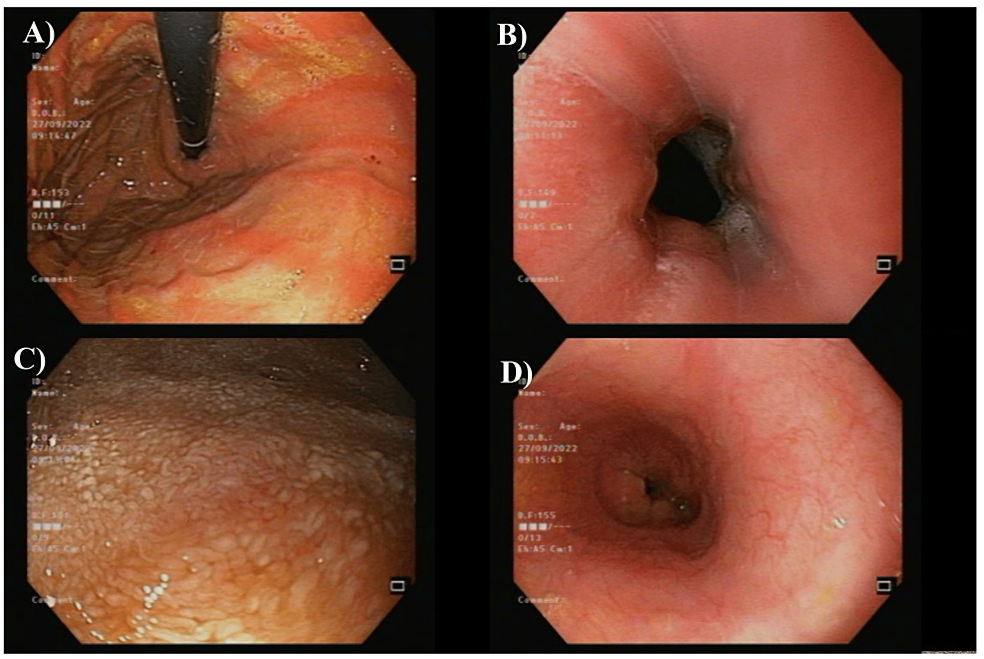
The first esophagogastroduodenoscopy (EGD) scanning of the studied case A: normal esophagus; B, D: the duodenum with scattered white patches; C: the stomach with a diffuse liner mucosal erythema

The upper and lower endoscopy were requested to investigate abnormal features of the gastrointestinal system. The esophagogastroduodenoscopy (EGD) scanning showed the esophagus with normal mucosa, while the stomach had a diffuse liner mucosal erythema with a significant amount of bile. No masses, polyps, or active bleeding were seen. The duodenum had scattered white patches with decreased fold number and mild notching of folds (Figure 3). Two biopsies were taken from the stomach and duodenum to identify any activity of Helicobacter pylori and define celiac disease, respectively.

**Figure 4:**
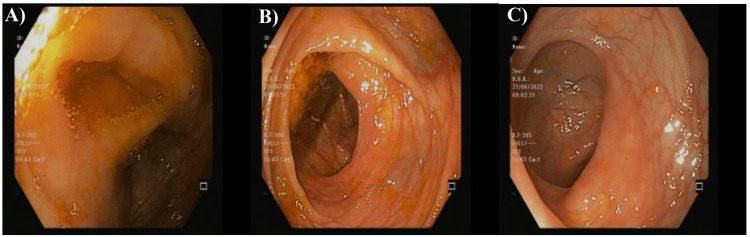
The first colonoscopy showed normal findings A: ileocecal valve; B: caecum; C: rectum

The colonoscopy didn't reveal any abnormalities in the rectum, terminal ileum, ileocecal valve, cecum, or ascending, transverse, descending, and rectosigmoid colon (Figure 4). The diagnosis at that step was pan-gastritis and duodenitis with a picture of celiac and normal ileo-colonoscopy. The patient was discharged at that step on vitamin B-12 supplements with follow-up in the clinic.

The duodenum biopsy showed intact intestinal villi, mild chronic inflammation without any identification of increased eosinophils, dysplasia, granuloma, or malignancy. The gastric biopsy showed mild chronic gastritis without any intestinal metaplasia/dysplasia or H. Pylori activity, which were not identified on the Giemsa stain.

The patient was still suffering from the same conditions, so she was admitted again on December 20, 2022, under general surgery as a case of intestinal obstruction. She was complaining of vomiting, constipation, and abdominal distension for one week. The laboratory investigations were performed and showed similar findings, as previously mentioned (Table 2). In addition, the white blood cell (WBC) count was slightly higher than before (12.17×109/L). The blood analysis of the heart enzymes showed a reduction in the levels of creatine kinase (23 U/L), an increase in the levels of troponin 1 (0.9 ng/mL), and normal values for lactate dehydrogenase (LDH) and creatine kinase-myocardial band (CK-MB).

**Table 2:** The second blood analysis of the studied case

Variables	Results	Normal values
Hematology		
White blood cells (WBC), ×10^9^/L	12.17 ↑	4-11
Hemoglobin (Hb), g/dL	11.9 ↓	12-15.1
Hematocrit (HCT), %	43	38-52
Platelets count (Plt), ×10^9^/L	590 ↑	150-400
Mean corpuscle volume (MCV), fl	63 ↓	80-100
Liver function and comprehensive metabolic panel (CMP)		
Magnesium (Mg), mmol/L	0.88	0.65-1.05
Albumin (Alb), g/L	40	35-52
Total bilirubin (Tb), µmol/L	6.5	5.1-20.5
Alanine transaminase (ALT), U/L	17	7-55
Aspartate transaminase (AST), U/L	23	8-48
Alkaline phosphatase (ALP), U/L	58	32-91
Gamma-glutamyl transpeptidase (GGT), IU/L	12	11- 55
Prothrombin time (Pt), seconds	15.8 ↑	11-13
Activated partial thromboplastin time (aPTT), seconds	30.8	21-35
International normalized ratio (INR)	1.2 ↑	<1.1
Kidney functions and basic metabolic panel (BMP)		
Sodium (Na), mmol/L	139	136-144
Potassium (K), mmol/L	4.4	3.6-5.1
Calcium (Ca), mmol/L	2.43	2.2-2.7
Chloride (Cl), mmol/L	106	96-106
Serum creatinine (sCr), µmol/L	42 ↓	53-97.2
Blood urea nitrogen (BUN), mmol/L	3.05	2.1-8.5
Phosphorus (Ph), mg/dL	1.2 ↓	2.5-4.5
Blood enzymes		
Lactate dehydrogenase (LDH), U/L	273	140-280
Creatine kinase (CK), U/L	23 ↓	30-135
Creatinine kinase-myocardial band (CK-MB), %	3.8	3-5
Troponin 1 (Trop-1), ng/mL	0.9 ↑	0-0.04

The CT and MRI were repeated in December 2022, and as compared with previous scanning, they didn't reveal any significant changes. A small bowel dilatation and air-fluid level with minimal fluid in between loops were still significant observations. Both kidneys were normal without any back-pressure changes.

**Figure 5:**
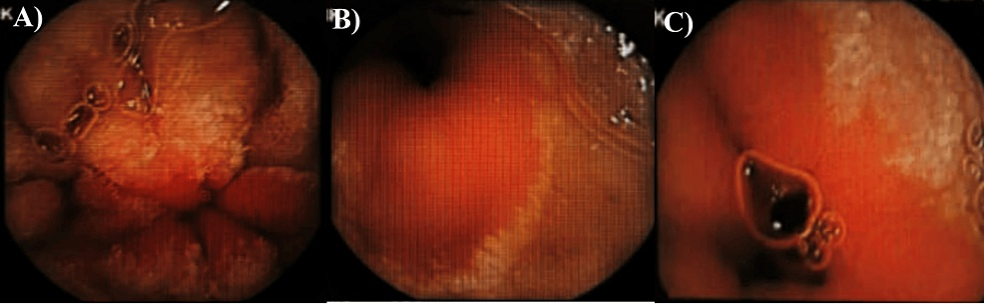
An abdominal capsule endoscopy of the studied patient An abdominal capsule endoscopy showed a small bowl segment with loss of villi and diaphragmatic stenosis, mostly in the jejunum. A: stenosis in the jejunum; B,C: small bowl erosion

Capsule endoscopy was performed and showed a small bowl segment with loss of villi and diaphragmatic stenosis, mostly in the jejunum. The recording ended without reaching the cecum (Figure 5). That was diagnosed as an NSAID-induced small bowel diaphragmatic stricture with loss of villi in the jejunum.

**Figure 6:**
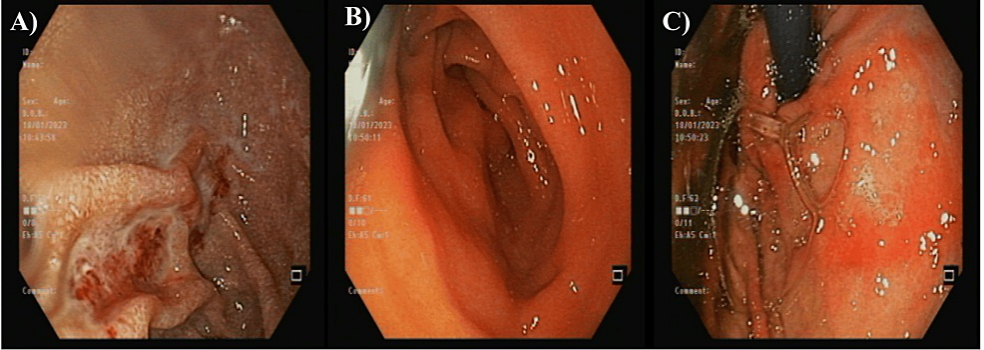
The second esophagogastroduodenoscopy (EGD) scanning of the studied case A: normal esophagus; B: the stomach with diffuse mucosal erythema with normal pylorus; C: duodenum had a scar

The patient was exposed to an open laparotomy to remove most of the strictures and stenosis. The intraoperative enteroscopy, which was entered through a surgical enterostomy, showed multiple strictures and ulcerations in the small bowel, mostly in the jejunum. She underwent bowel resection for the most stenotic strictures with two end ileostomies, where 16 cm from the ileum was removed. The pathology was an acute inflammatory process, which might be due to the early phase of acute inflammatory bowel disease, or the other differential includes microorganisms and drug-induced inflammatory processes, among others. The second EGD showed a normal esophagus, while the stomach had a diffused mucosal erythema with a normal pylorus. The duodenum had a remarkable scar on the third part of the duodenum (Figure 6).

**Figure 7:**
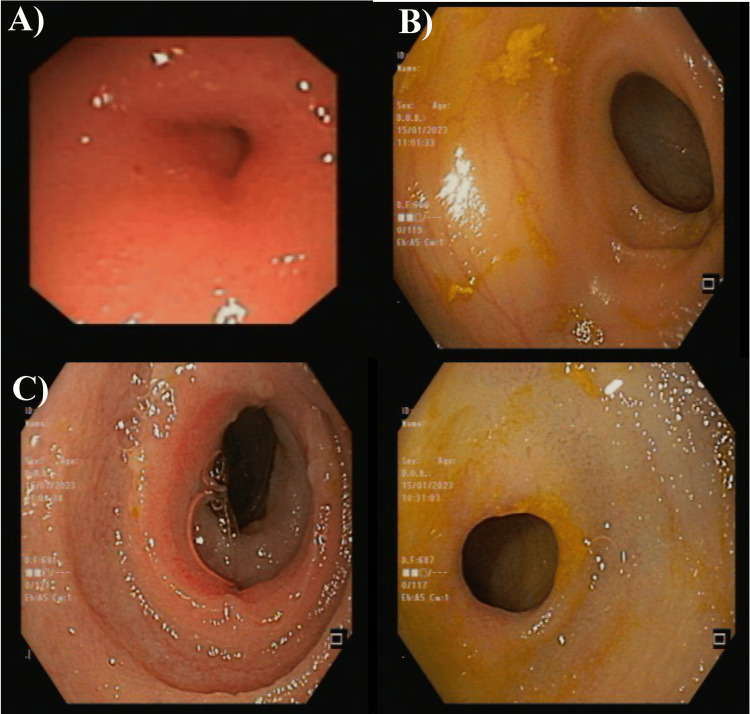
The intraoperative enteroscopy of the studied case The capsule endoscopy showed multiple strictures and ulcerations in the small bowel, which are of the small intestinal diaphragms. Images taken after surgery showed multiple strictures with normal mucosa. A: the left stoma with mild diaphragmatic stenosis and normal mucosa; B-D: the right stoma had a small bowel with three areas of diaphragmatic-like stricture

During the above-mentioned procedure, the enteroscopy was done through the stoma and showed a proximal small bowel of the colostomy (left stoma) with mild diaphragmatic stenosis seen at five and 10 cm from the ileostomy site with normal mucosa. The ileostomy (right stoma) had a small bowel with three areas of diaphragmatic-like stricture seen at five, nine, and 12 cm from the stoma with normal mucosa. The endoscopy diagnosis was reported as a diffused erythema in the third part of the duodenum and multiple small bowel diaphragmatic stenosis and strictures in the proximal and distal small bowel. That might be due to excessive use of NSAIDs (Figure 7).

**Figure 8:**
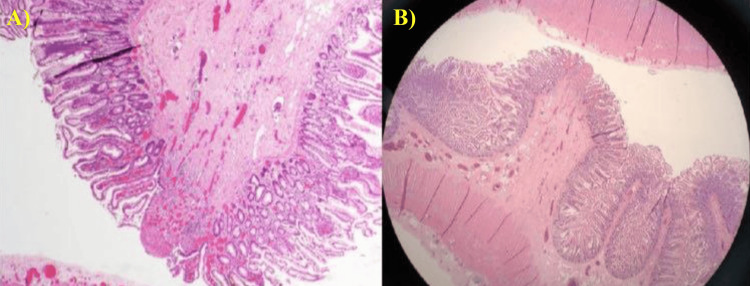
Histopathological analysis of the duodenum biopsy of the studied case, which showed features of small intestinal diaphragm disease The biopsy was taken within the second EGD. The slide was stained with hematoxylin and eosin (H&E) stains and visualized by Leica DM 2000 microscopy (Leica Microsystems, Danaher Corp., Washington, US). A: high magnification of 100×; B: low magnification of 20× EGD - esophagogastroduodenoscopy

During the second esophagogastroduodenoscopy (EGD), a biopsy was obtained from the duodenum. The findings indicated loose fibrous tissue at the mucosal base, along with multiple vessels and eosinophils present. Additionally, a nodular lesion was observed in the submucosa, adjacent to the club-shaped tip of a diaphragm, with smooth muscle converging towards the ulcer's base. The muscularis mucosae was intertwined with the submucosal lesion. It's noteworthy that there was relatively minimal inflammation with non-specific chronic duodenitis on both sides of the ulcer. The biopsy was negative for any significant villous architectural changes, intraepithelial lymphocytosis, active duodenitis, granulomas, dysplasia, or malignancy (Figure 8). The microscopic images revealed some features of the acute inflammatory process, which might be due to an early phase of acute inflammatory bowel disease.

Two days post-surgery, a follow-up CT was performed and revealed a resolution of the previously seen intestinal obstruction. There was a stationary course for the other changes that had previously been seen. Inflammatory changes were still seen at the operative site in the right iliac fossa with minimal fluid. A well-opacified bowel loop and contrast were seen reaching the rectum with no sign of obstruction. The liver, intra- and extra-hepatic biliary radicles, gallbladder, pancreas, spleen, and both kidneys are normal. After surgical relief of obstruction, the patient gained weight and improved dramatically, as observed by the regular follow-up in the clinic. The patient was advised to decrease the frequency and duration of the use of NSAIDs and to replace them with paracetamol.

## Discussion

It has been theorized that NSAIDs induce small bowel enteropathy by disrupting mucosal and cellular integrity. These drugs, functioning as lipid-soluble weak acids, interfere with enterocyte phospholipid membranes, leading to uncoupling of mitochondrial phosphorylation and eventual loss of mucosal barrier function [10]. Additionally, NSAIDs are implicated in compromising villous blood flow through cyclooxygenase-mediated mechanisms. Although rare, diaphragm strictures are considered pathognomonic for NSAID usage. Typically found in the small intestine, these strictures consist of multiple, thin septa measuring 2-3 mm in thickness, which can significantly reduce intestinal lumen size, potentially resulting in bowel obstruction [9].

In the current study, NSAID-induced damage in the small intestine manifests as ulceration, strictures, and enteropathy. The results of routine endoscopic evaluations of the stomach, duodenum, and colon were often negative in this setting and did not predict the presence or absence of small bowel lesions. In addition, the radiographic assessment of the small intestine is usually not diagnostic. The patient underwent endoscopic examinations, revealing pangastritis and duodenitis with features suggestive of celiac disease. Biopsies from the stomach and duodenum showed mild chronic gastritis and non-specific chronic duodenitis, respectively, without evidence of Helicobacter pylori infection or significant pathology. Despite vitamin B-12 supplementation, the patient continued to experience symptoms consistent with irritable bowel syndrome (IBS). Subsequent admission for intestinal obstruction led to laparotomy, revealing multiple strictures and ulcerations in the small bowel, likely due to excessive NSAID use.

A capsule endoscopy confirmed small bowel diaphragmatic stenosis, prompting bowel resection without obstruction. Follow-up imaging showed the resolution of obstruction with persistent inflammatory changes. Overall, the patient's condition highlights the challenges of diagnosing and managing gastrointestinal complications, especially in the context of NSAID-related injuries and concurrent conditions like IBS.

In a previous case study, a 45-year-old American female was diagnosed with small bowel obstruction, and she complained of recurrent small intestinal obstruction caused by NSAID-induced small bowel strictures. She was diagnosed with capsule endoscopy and managed by strict plastic surgery [11]. Another report of three cases who were diagnosed with NSAID-induced diaphragm disease showed that the capsule endoscopy showed circumferential strictures of the ileum, which were managed by segmental resection and enterotomy [12]. Another case of a 60-year-old Irish male was diagnosed with multiple intestinal diaphragms unmasked with severe duodenal ulcers that resulted from long-term exposure to 5-aminosalicylic acid (5-ASA) and immune suppression and was investigated by abdominal endoscopy and CT scanning [13]. Unlike our results, a case of a 62-year-old male presented with recurrent periumbilical pain and small bowel obstruction, which led to exploratory laparotomy revealing jejunal submucosal fibrosis consistent with NSAID-induced enteropathy [14]. All of these case reports are in compliance with our reported results, which highlight the diagnostic challenge of NSAID-induced diseases and occasionally necessitate laparotomy for definitive diagnosis in inconclusive preoperative assessments.

## Conclusions

Patients who are on long-term NSAID therapy should be monitored closely for gastrointestinal complications, including the development of small intestinal diaphragms. Management strategies may include discontinuation or dose reduction of NSAIDs and consideration of alternative pain management options. Additionally, patients with known small intestinal diaphragms may require endoscopic evaluation and possible surgical intervention, depending on the severity of their symptoms and complications. A high index of suspicion and the judicious use of endoscopic techniques, including push enteroscopy and intraoperative enteroscopy, permitted an accurate diagnosis in many cases. These lesions should be considered in any patient who has taken NSAIDs and presents with acute obscure gastrointestinal hemorrhage, small bowel obstruction, or chronic occult gastrointestinal blood loss and iron-deficiency anemia.
